# Onset and transition of and recovery from adverse development: Study methodology

**DOI:** 10.1111/eip.12882

**Published:** 2019-11-05

**Authors:** Johanna T.W. Wigman, Gerdina H.M. Pijnenborg, Richard Bruggeman, Maarten Vos, Anita Wessels, Inez Oosterholt, Maaike Nauta, Renee Stelwagen, Lana Otto, Anniek Wester, Lex Wunderink, Esther Sportel, Nynke Boonstra

**Affiliations:** ^1^ University of Groningen, University Medical Center Groningen, Rob Giel Research Centre (RGOc) University of Groningen Groningen The Netherlands; ^2^ Department of Psychology University of Groningen Groningen The Netherlands; ^3^ GGZ (Mental Health Organization) Drenthe Assen The Netherlands; ^4^ Mediant Mental Health Organization Enschede The Netherlands; ^5^ Dimence Mental Health Organization Deventer The Netherlands; ^6^ Accare Youth Mental Health Organization Groningen The Netherlands; ^7^ Mental Health Organization Friesland Leeuwarden The Netherlands

**Keywords:** ARMS, clinical staging, early intervention, OnTheROAD, protocol

## Abstract

**Aim:**

Early intervention programs for first‐episode psychosis have led to the awareness that the period *before* onset of a first episode is important in light of early intervention. This has induced a focus on the so‐called ‘at risk mental state’ (ARMS). Individuals with ARMS are at increased risk for later psychotic disorder, but also for other psychiatric disorders as well as poor psychosocial functioning. Thus, adequate detection and treatment of ARMS is essential.

**Methods:**

Since 2018, screening for and treatment of ARMS is recommended standard care in the Netherlands. Implementation is still ongoing. We initiated a naturalistic long‐term cohort study of ARMS individuals, the onset and transition of and recovery from adverse development (OnTheROAD) study, with the aim to monitor course and outcome of symptoms and psychosocial functioning over time, as well as patterns of comorbidity and associations with factors of risk and resilience. To this end, participants complete a broad battery of instruments at baseline and yearly follow‐up assessments up to 3 years. Outcome is defined in terms of symptom severity level, functioning and quality of life. In particular, we aim to investigate the impact of negative symptoms as part of the ARMS concept. Results from this study can aid in refining the existing ARMS criteria, understanding the developmental course of ARMS and investigating the hypothesized pluripotentiality in outcome of ARMS. New knowledge may inform the further development of specialized early interventions.

**Results and Conclusions:**

In this article, we describe the rationale, outline and set‐up of OnTheROAD.

## 1 INTRODUCTION

Clinicians and researchers are still searching for valid diagnostic tools to select optimal interventions and accurately predict course and outcome of early psychopathological expressions (Kapur, Philips, & Insel, 2012). The current classification system based on the diagnostic and statistical manual of mental disorders (DSM‐5, American Psychiatric Association, 2013) has, similarly to its predecessors, shortcomings in these respects (Frances & Widiger, 2012; Kendell & Jablensky, 2003; Kendler, Zachar, & Craver, 2011). Therefore, a different perspective on psychopathology is needed, recognizing that psychological symptoms do not keep to the boundaries of diagnostic categories, do not emerge out of the blue but often develop from precursor stages, and vary greatly between individuals (McGorry, 2007; McGorry, Hickie, Yung, Pantelis, & Jackson, 2006; McGorry & van Os, 2013). In recent years, the concept of clinical staging was introduced (McGorry et al., 2006), promoting a subtler, more differentiated addition to the diagnostic process, studying the development of psychopathological processes in individuals. The fundamental idea of this model, developed in the context of psychosis, is that different stages of psychopathological development (ie, with increasing psychopathological severity) can be distinguished that require different types of interventions that are effective specifically in that stage (McGorry et al., 2006).

Psychotic disorders are considered among the most severe mental disorders, in terms of both individual and societal burden (van Os & Kapur, 2009). Therefore, early detection and treatment of psychosis should be highly prioritized (McCrone, Patel, Knapp, & Lawton‐Smith, 2008; McGorry, Killacky & Yung, 2007). Early intervention programs for first‐episode psychosis have led to the awareness that the period *before* onset of such a first episode is very important in light of early intervention. This period is often dubbed ‘prodromal phase’ retrospectively after onset of a psychotic episode (Yung et al, 2003). However, a broad line of research has shown that its clinical picture, characterized by psychological distress, attenuated psychotic symptoms (APS) and a broad spectrum of other psychiatric symptoms, can also be identified as a prospective risk factor (Yung et al, 2003). From this perspective, it is labelled rather as ultra high risk phase, clinical high risk phase or at risk mental state (ARMS), indicating that, although this population is at risk for developing more severe illness, adverse development is not necessarily unavoidable.

Initially, ARMS was mainly investigated as predictor of later psychotic disorder, with about 36% of the ARMS population developing a first psychotic episode within 3 years of follow‐up (Fusar‐Poli et al., 2012). There is an ongoing discussion on the predictive specificity of ARMS. Although ARMS has been shown to be specific in its prediction of later psychosis (Fusar‐Poli et al., 2017; Woods et al., 2018), it has also been suggested that ARMS has additional importance as a predictor for a broader spectrum of adverse development in terms of both (persistent) non‐psychotic symptomatology and impaired functioning (Yung et al., 2012), stressing the suggested pluripotent nature of ARMS (McGorry, Hartmann, Spooner, & Nelson, 2018). This pluripotentiality‐hypothesis implies that earlier expressions of psychopathology can be transient, persist or develop into a variety of clinical disorders (McGorry et al., 2018). For example, early psychotic symptoms have been shown to predict the development of later psychotic disorder (Poulton et al., 2000; Welham et al., 2009), but also of other later (eg, mood) disorders (Addington et al., 2011; Fusar‐Poli et al., 2012; Kaymaz et al., 2012; Lin et al., 2015; McGrath et al., 2016; Werbeloff et al., 2012) and/or impaired psychosocial functioning (Addington et al., 2011). Part of these complex associations may be explained by the fact that, although the definition of ARMS currently relies heavily on positive psychotic symptomatology, presence of other symptoms (eg, anxiety, depression) is very common (Yung et al, 2007; Lin et al., 2015). In addition to serving as an indicator of severe mental health problems, early psychotic symptoms are also related to current and future poor functional outcome (Cotter et al., 2014, 2018). Both types of outcome are equally important, but are not necessarily identical: functional impairments can occur without noticeable symptomatic impairments and vice versa (Lin, Wood, & Yung, 2013; Verma, Subramaniam, Abdin, Poon, & Chong, 2012; Wunderink, Sytema, Nienhuis, & Wiersma, 2009).

To better understand the nature and course of ARMS over time as well the factors that may impact on this course, a broader assessment of the clinical presentation in terms of both symptomatology and functioning is needed. Individual risk profiling within this broader picture might help differentiate between individuals at highest risk of poor outcome and individuals with highest chance of recovery. Since 2018, screening for as well as monitoring and treatment of ARMS are included in the recommended standard care in the Dutch mental health care system. We have been successful in implementing these new procedures in the North of the Netherlands and are now setting up a study to follow a cohort of individuals identified according to these new procedures: the Onset and Transition of and Recovery from Adverse Development (OnTheROAD) study. This project is in line with other initiatives to follow cohorts of individuals at ARMS (see eg, Brewer et al., 2006; Deriu, Moro, & Benoit, 2018 for overviews of such cohorts). The regular guidelines are limited almost exclusively to positive symptoms of psychosis and functioning. In OnTheROAD, the goal is to assess individuals with ARMS from a broader perspective, capturing multiple domains of psychopathology, functioning, and factors of risk and resilience. In particular, we are interested in the role of negative symptoms in ARMS (Wunderink, 2017), as these symptoms are increasingly acknowledged as important predictors of both clinical (Pisculic et al., 2012; Demjaha, Valmaggia, Stahl, Byrne, & McGuire, 2010) and functional (Kim et al., 2013; Lin et al., 2011; Yung et al, 2019) outcome. The specific aim is to investigate the added value of negative symptoms as a possible extension of current ARMS criteria. Broadening the set of clinical measures and factors of risk and resilience that may determine outcomes of ARMS enables individual risk profiling and the investigation of the hypothesized pluripotentiality of ARMS. This article outlines the rationale, outline and methodological set‐up of the On The ROAD study.

## 2 METHODS

### 2.1 Design

The design of the study is a naturalistic cohort study of individuals with ARMS. The present study is implemented in multiple mental health care centres. The main research centre is the Rob Giel Research center (RGOc) in Groningen, the Netherlands, a collaborative research centre of six large mental health care organizations (MHOs) in the North‐East of the Netherlands. A pilot phase of OnTheROAD started in January 2016. During the first period (2016‐2018), the main focus was on setting up and implementing the infrastructure for the clinical part of the Early Detection project (screening, interview and treatment). In 2019, the official study period for the additional test battery started.

### 2.2 Sample

To meet inclusion criteria, individuals need to be aged between 14 and 35 years, newly referred to one of the mental health care institutes of the participating centres in the North‐East of the Netherlands (MHO Friesland, MHO Drenthe, Dimence Group, Mediant, University Centre Psychiatry, MHO Lentis and Accare) for the treatment of (non‐psychotic) mental health problems, meeting ARMS criteria and having provided informed consent. Exclusion criteria are a diagnosis of a current psychotic disorder according to the DSM, being unable to fill out questionnaires and limited command of the Dutch language.

### 2.3 Procedure

All new patients aged 14‐35 are routinely screened online for precursor stages of psychotic symptoms with the prodromal questionnaire‐16 (PQ‐16; Ising et al., 2012). Outcome of the screening procedure does not influence decisions regarding standard care for other, non‐psychotic mental health complaints. In case of a sum score ≥ 6, the Comprehensive Assessment of At risk Mental States (CAARMS, Yung et al., 2005) and the Social and Occupational Functioning Assessment Scale (SOFAS, American Psychiatric Association, 1994) are assessed to determine ARMS. Based on the CAARMS interview in combination with the SOFAS, each participant is assigned to one of these three categories:

1. No high risk, no first episode of psychosis
2. ARMS
3. First episode of psychosis

Patients in category 1 continue their regular treatment. Patients in category 3 are referred to a first‐episode treatment program. Category 2 is the target population of OnTheROAD. Individuals with ARMS are offered evidence‐based care (including monitoring and treatment) in the form of an add‐on module on top of their regular treatment. This evidence‐based module is based on cognitive behavioural therapy (CBT) (French & Morrison, 2004) and is tailored to and routinely offered to individuals with ARMS. This intervention has been shown to result in 50% reduction of the number of transitions to psychosis (from 20% to 10%) (Van der Gaag et al., 2012; Van der Gaag et al., 2013) and has been shown to be very cost‐effective (Ising et al., 2015).

The ARMS category consists of three subgroups: a group with (a) APS, (b) brief limited psychotic symptoms (BLIPS) (ie, full‐blown psychotic symptoms that resolve spontaneously within a week) and (c) schizotypal personality or a first‐degree relative with psychotic history, in combination with a drop in functioning (Nelson, Yuen & Yung, 2011; Yung et al, 1996). All three subgroups are included in OnTheROAD.

After identification of ARMS status, participants are invited to take part in OnTheROAD by a research assistant during the meeting where the CAARMS results are discussed. If interested, participants sign a written consent form. A link to the self‐report questionnaires is then sending to the participant via email; interviewer‐rated instruments are assessed during a face‐to‐face contact moment. The decision whether or not to enter OnTheRoad does not have any influence on the type of treatment that the participant receives or on any other variables. In the first stage of the project, results are not shared with participants or clinicians who are treating them. After collecting data of N = 100 participants, to aim is provide personal reports with the scores of the individual participant compared to the group level scores of the N = 100 sample that the clinician can discuss with the participant.

Standard care is offered to all participants, regardless of whether they enter OnTheRoad or not. Those who do enter OnTheROAD are invited to complete an extra assessment battery consisting of several self‐report questionnaires and interviewer‐rated instruments that are described in section 3.

### 2.4 Ethics

Because OnTheROAD does not intervene in regular treatment, the study was exempted by the Medical Ethical Committee of the University Medical Centre Groningen (M15.173558). Written informed consent is asked from all participants older than 18 years for the use of the collected clinical data. For participants between the age of 14 and 18, written informed consent is asked from both youngster and parents.

## 3 INSTRUMENTS

### 3.1 Clinical measures

Both categorical (yes/no diagnosis) and dimensional (continuous scores consisting of sum scores of all individual items) of multiple psychopathological domains are collected:

#### 3.1.1 Clinical diagnosis

The mini‐SCAN interview, a structured clinical diagnostic interview (Nienhuis, van de Williger, Rijnders, de Jonge, & Wiersma, 2010), is assessed by trained research assistants in a face‐to‐face interview. The mini‐SCAN is a validated (Nienhuis et al., 2010) short version of the Schedules for Clinical Assessment in Neuropsychiatry (SCAN) (Wing et al., 1990), covering a wide range of DSM diagnoses. All disorders of which criteria are met are listed as output at the end of the interview.

#### 3.1.2 Psychotic symptoms

Psychotic symptoms are assessed in a two‐step procedure: first, the PQ‐16 (Ising et al., 2012) is completed. The PQ‐16 consists of 16 self‐rated items that are rated on a two‐point scale (true/false) (14 positive psychotic symptoms and 2 negative symptoms). Items are summed. The PQ‐16 showed good concurrent validity with the interview‐based CAARMS diagnoses. Using a cut‐off score of six or more symptoms, Ising et al. (2012) found a high true positive rate (87%) and high specificity (87%) when differentiating UHR/psychosis from those with no CAARMS diagnosis.

When scoring above the pre‐set cut‐off score of ≥6, the Positive Symptom Scale of the CAARMS (Yung et al., 2005) interview is assessed. The CAARMS is a semistructured interview, developed specifically to determine if an individual meets criteria for ARMS or for onset of first psychotic disorder, based on assessment of the intensity/severity, frequency/duration, and fluctuation of APS over the past 12 months. The positive symptom scale that was used consists of four subscales: (a) unusual thought content; (b) non‐bizarre ideas; (c) perceptual abnormalities; and (d) disorganized speech. Scores for each subscale are rated on intensity, frequency and duration, pattern of symptoms and level of distress. The CAARMS has good psychometric properties (Yung et al., 2005).

#### 3.1.3 Negative symptoms

Negative symptoms are assessed with the Brief Negative Symptom Scale (BNSS; Kirkpatrick et al., 2011). The BNSS consists of 13 items that are rated by an interviewer on six subscales (blunted affect, alogia, asociality, anhedonia and avolition). All items are rated on a seven‐point scale. The BNSS has good psychometric properties as it has shown high interrater consistency (intraclass correlation coefficient [ICC] = 0.96), test‐retest consistency (*r* = 0.81 over 1 week) and internal consistency (alpha = 0.93; all values based on total score). In addition, associations with instruments assessing positive symptoms and other instruments assessing negative symptom established the discriminant and concurrent validity of the BNSS (Kirkpatrick et al., 2011).

#### 3.1.4 Mood, Anxiety and Stress

Mood, anxiety and stress are assessed with the Depression, Anxiety and Stress Scale‐21 (DASS‐21; Lovibond & Lovibond, 1995). The DASS‐21 consists of 21 self‐reported items (seven per domain), rated on a four‐point scale. The DASS‐21 has good psychometric properties in terms of factorial structure, internal consistency and concurrent validity (Antony, Bieling, Cox, Enns, & Swinson, 1998).

#### 3.1.5 Mania

Mania is assessed with the self‐reported Altman Self‐Rating Mania Scale (ASRM; Altman, Hedeker, Peterson, & Davis, 1997). The ASRM contains five items covering several symptom domains of mania (elevated/euphoric mood, increased self‐esteem, decreased need for sleep, pressured speech, and psychomotor agitation). For each item, five possible statements are given on a five‐point range that represent increasing levels of mania. The ASRM has shown good psychometric properties in clinical samples, with good test‐retest reliability on a sample of depressed and manic patients, the ability to assess severity of manic symptoms in patients with mania and to pick up change following treatment (Altman et al., 1997).

#### 3.1.6 Eating disorders

Symptoms of eating disorders are assessed with the SCOFF, a five item self‐report questionnaire that screens for eating disorders (Morgan, Reid, & Lacey, 1999). The SCOFF addresses core features of anorexia nervosa and bulimia nervosa: (a) feeling sick or vomiting after eating; (b) losing control about the amount of feed one eats; (c) losing more than one stone in 3 months, (d) believing yourself to be fat and (e) food dominating your life. Items are scored as yes/no. High levels of reliability and acceptable trade‐offs between sensitivity and specificity have been found for the SCOFF in the original as well as translated versions (Botella, Sepúlveda, Huiling, & Gambara, 2013).

#### 3.1.7 Problematic behaviour

Aggression and self‐harm are assessed using an instrument that was developed for the European Long‐acting Antipsychotics in Schizophrenia Trial study. Three questions were developed, based on other subscales of several other questionnaires, being the Staff Observation Aggression Scale‐Revised (Nijman et al., 1999), the Modified Overt Aggression Scale (Kay, Wolkenfield, & Murril, 1988) and the Self Harm Behaviour Questionnaire (Gutierrez, 1998). These questions cover whether, during the past month, the participant (a) had deliberately harmed oneself, (b) had been involved in a violent incident or had been a victim of violence or (c) had attacked somebody oneself. Formal psychometric information is not yet available.

#### 3.1.8 Somatization

Symptoms of somatization are assessed with the SPHERE‐12 (Hickie et al., 2001), that included 12 self‐report items from the original 34‐item Somatic and Psychological Health Report (SPHERE) questionnaire. The SPHERE‐12 covers six somatic (fatigue, somatic complaints) and six psychological (depression, anxiety) items on a three‐point Likert scale. Combining the somatic and psychological dimensions can help to identify those patients with problems on one of these domains, on neither or on both. This system has shown to have acceptable validity and reliability (Hickie et al., 2001).

#### 3.1.9 Alexithymia

Alexithymia, or the inability to identify and describe emotions adequately, is assessed with the Toronto Alexithymia Scale (TAS‐20; Bagby, Parker, & Taylor, 1994). The TAS‐20 consists of 20 self‐report items, subdivided into three subscales: difficulty with describing feelings (five items), difficulty with identifying feelings (seven items) and externally‐oriented thinking (eight items), all rated on a five‐point Likert scale. The TAS‐20 was shown to have good internal consistency and test‐retest reliability, as well as a three‐factor structure that matches with the alexithymia construct (Bagby et al., 1994).

#### 3.1.10 Clinical Global Impression

The Clinical Global Impression‐Severity scale (CGI‐S; Guy, 1976) is used to assess overall severity of illness on a seven‐point scale. The interviewer rates the severity of the patient's illness at the time of assessment, relative to their previous experience with similar patients. The CGI was shown to have good internal consistency and concurrent validity in a clinical sample (Leon et al., 1993).

### 3.2 Functioning

#### 3.2.1 SOFAS

Functioning is assessed using the Social and Occupational Functioning Scale (SOFAS; APA, 1994). The SOFAS is an interview‐rated scale that gives a global assessment of the level of social and occupational functioning. Scores can range between 0 (not functioning at all) and 100 (superior functioning). In scoring the SOFAS, impact of symptoms is taken into account; therefore, this measure reflects a combination of symptomatic and functional outcomes. The lowest score in the past year is used in the current study.

#### 3.2.2 Global functioning scales

The Global functioning scales (Cornblatt et al., 2007) comprise two interviewer‐rated scales that assess functioning specifically in the ARMS population: the Global Functioning Social (GF: Social) and the Global Functioning Role (GF: Role) scales. The two scales are designed along the lines of the GAF and SOFAS scales, but measure these two sub‐domains separately. In addition, the scales take age and phase of illness into account. Both scales can be rated on a scale from 1 (severely disabled) to 10 (superior functioning) with each score described by an anchor. Both scales showed high interrater reliability and sensitivity to change and preliminary support for construct validity was also reported by Cornblatt et al. (2007).

### 3.3 Background factors

#### 3.3.1 Demographics

The following demographic information is obtained through self‐report: age, gender, ethnicity, relationship status, living arrangements, education, employment and sexual orientation.

#### 3.3.2 Potential risk factors

#### 3.3.3 Bonding

Bonding is assessed with the inventory for parent and peer attachment (IPPA; Armsden & Greenberg, 1987), a 48‐item self‐report questionnaire that asks about bonding to the participant's mother (or mother figure), father (or father figure) and significant other (16 items per person). Items are rated on a five‐point Likert scale. The IPPA has shown to have good internal consistency, test‐retest reliability and good concurrent and divergent validity (Armsden & Greenberg, 1987).

#### 3.3.4 Life events

Life events are assessed using the List of Threatening Experiences (LTE; Brugha, Bebbington, Tennant, & Hurry, 1985), a self‐report questionnaire that asks about 12 potential life events that may have happened during the past year, for example, having experienced serious illness or loss and that are scored as yes/no. In a clinical population, the LTE was shown to have high test‐retest reliability and also good agreement with information from an external informant. Good concurrent validity was shown with a semi‐structured life events interview (Brugha & Cragg, 1990).

#### 3.3.5 Trauma

Youth trauma is assessed using the Dutch version of the childhood trauma questionnaire (CTQ; Bernstein et al., 1994). The CTQ is a 28‐item self‐report instrument that assesses the experience of five types of youth trauma (emotional abuse, physical abuse, sexual abuse, emotional neglect and physical neglect). The extent to which each type of trauma has been experienced is rated on a five‐point Likert scale. The CTQ has shown high internal consistency, good test‐retest reliability (interval 2‐6 months) and good concurrent validity (Bernstein et al., 1994).

#### 3.3.6 Discrimination

To assess discrimination, the same items are assessed as in the Transitions study (Purcell et al., 2015), who adapted three questions from a scale assessing discrimination in the Quality of Life in Newly Diagnosed Epilepsy Instrument (NEWQOL; Abetz, Jacoby, Baker, & McNulty, 2000) battery.

#### 3.3.7 Family history of mental disorder

Family history of mental disorder is assessed by inquiring whether the father, mother or sibling(s) of the participant ever had any psychiatric problems. If yes, further questions on the nature of these problems and whether professional treatment was sought are probed.

### 3.4 Cognitive functioning

#### 3.4.1 Neurocognition

Neurocognition is assessed using the Cambridge Neuropsychological Test Automated Battery (CANTAB, 2017; www.cantab.com). The CANTAB is a computerized battery of tests that screens several relevant cognitive domains: memory (verbal, working and visual), spatial planning, strategy, attention flexibility, alertness and motor speed. This often‐used battery has shown to be able to adequately discriminate between healthy adults and individuals with psychiatric disorders (Egerhazi, Berecz, Bartok, & Degrell, 2007; Haring, Mottus, Koch, Trei, & Maron, 2015).

#### 3.4.2 Social cognition

Social cognition is assessed using the Faux Pas (Stone, Baron‐Cohen, & Knight, 1998). The Faux Pas presents the participant with nine vignettes describing social situations. The participant is then asked to answer several written questions to investigate whether they recognized the faux pas in the story. The Faux Pas has shown excellent reliability in a Swedish sample (Söderstrand & Almkvist, 2012).

### 3.5 Quality of life

Following the Purcell et al. (2015) transitions study, who, in turn, followed Murphy, Herrman, Hawthorne, Pinzone, and Evert (2000), quality of life is assessed with one item from the WHOQOL‐100 where participants rated their overall quality of life during the past 4 weeks on a five‐point scale.

## 4 FOLLOW‐UP PROCEDURE

All measures described above are assessed at baseline. The CAARMS and the SOFAS are then assessed every 3 months for 1 year, following standard procedures for treatment of ARMS. Participants are invited for follow‐up assessments after 1, 2 and 2 years when all measurements are re‐assessed.

## 5 STATISTICAL ANALYSES

Analyses include *t*‐test, Chi‐square, Pearson/Spearman correlations, multiple linear regression and multiple logistic regression. Survival analysis will be used to predict the onset of psychotic disorder and other mental disorder, controlling for relevant covariates (including gender, age, severity of psychopathology, history of mental health care, familial history of psychopathology). Linear regression will be used to predict psychosocial outcome, controlling for relevant covariates. Multinomial logistic regression will be used for more detailed analyses, such as predicting different categories of functional outcomes (eg, working, voluntary activities, household occupations). Dimensional assessments of psychopathology will be transformed when necessary due to non‐normality. Beta coefficients, ORs and 95% confidence intervals will be calculated.

## 6 DISCUSSION

This article describes the research protocol of OnTheROAD, a study in young people at risk for severe mental illness, namely individuals with ARMS. Although the predictive specificity of ARMS remains a topic under debate, ARMS is considered a risk factor for (a) later psychotic disorder, (b) many other psychiatric disorders and (c) poor psychosocial functioning. Therefore, broader assessment of the developmental course and outcome of ARMS over time is necessary. The objective of OnTheROAD is to follow a cohort of individuals with ARMS who receive state‐of‐the‐art care specific for ARMS, by monitoring the course of ARMS over time and, specifically, to assess individuals with ARMS from a broader perspective, by assessing multiple domains of psychopathology, functioning and factors of risk and resilience. In particular, we are interested in the role of negative symptoms in ARMS (Wunderink, 2017), in terms of both characterization of ARMS and their predictive value. Results of this study may aid in refining the existing ARMS criteria and developing more effective and personalized early interventions.

OnTheROAD joins a larger movement of monitoring ARMS over time, but also has several innovative aspects. Firstly, it assesses not only psychotic symptoms as predictors of outcome, but other potentially relevant symptoms as well. This addresses in more detail the heterogeneity of ARMS and fits the idea that risk factors can be pluripotential, predicting a wider range of poor outcome. Secondly, not only onset of first psychotic disorder is investigated. On the one hand, the focus on prediction of transition to psychotic disorders as primary outcome of the ARMS trajectory has been shown to be too narrowly defined (McGorry et al., 2018; Yung et al., 2012); on the other hand, recent studies again suggest more specificity of prediction (Fusar‐Poli et al., 2017; Woods et al., 2018). This study will contribute to the ongoing discussion on the specificity of ARMS for predicting clinical outcome. Thirdly, the study explores a broader range of conceivable predictors of clinical and functional outcome besides positive psychotic symptoms, in particular negative symptoms.

By means of OnTheROAD, we add to a broader development in the field that examines the pluripotentiality of ARMS. We aim to improve our understanding of the clinical picture of ARMS by taking a developmental, broader and transdiagnostic perspective and, eventually, we hope to improve clinical mental health care by providing more detailed information of individual patients' psychopathological profiles by combining insights from the clinical staging model (ie, the developmental stage of illness severity) with more personalized risk profiles based on context (ie, risk and protective factors, other patterns of co‐occurring psychopathology), so that provided care can be better matched to individual needs (Wunderink, 2018).

Recruitment of participants now takes place in mental health care services. In the future, we aim to extend recruitment also to General Practitioners, possibly using different strategies to screen sub‐populations at heightened risk (Boonstra, Wunderink, Sytema, & Wiersma, 2009). This step will also enable us to study earlier phases of the clinical staging model, as phases of developing mental illness that precede ARMS are then also captured.

## CONFLICT OF INTERESTS

None of the authors has any conflicts of interest.

## Data Availability

Data sharing not applicable ‐ no new data generated.
